# Cancer Risk in Nepal: An Analysis from Population-Based Cancer Registry of Urban, Suburban, and Rural Regions

**DOI:** 10.1155/2024/4687221

**Published:** 2024-07-10

**Authors:** Uma Kafle Dahal, Kopila Khadka, Kiran Neupane, Sandhya Chapagain Acharya, Anjani Kumar Jha, Pradip Gyanwali, Gehanath Baral

**Affiliations:** ^1^Nepal Health Research Council, Ramshah Path, Kathmandu, Nepal; ^2^Department of Clinical Oncology, National Academy of Medical Science, NAMS, Bir Hospital, Kathmandu, Nepal; ^3^Department of Radiation Oncology, Kathmandu Cancer Center, Tathali, Bhaktapur, Nepal; ^4^Singhania University, India

## Abstract

**Background:**

Cancer is one of the leading causes of death globally. The low and middle-income countries (LMICs) cover a major share of the global cancer burden; however, most of the LMICs including Nepal still lack national cancer control and prevention strategies. Since 1^st^ January 2018, the Nepal Health Research Council (NHRC) started the population-based cancer registry (PBCR) in urban, suburban, and rural regions to support evidence-based cancer control intervention in each geographical region.

**Methods:**

Data regarding incidence and mortality was collected by the PBCR in Nepal. Indirect and direct methods were used to collect data from health facilities and communities, respectively. Registered cases of incidence and mortality from 1^st^ January 2019 to 31^st^ December 2019 were used. Each case was verified for correctness and duplication followed by residence confirmation via phone call. Guidelines and principles of the International Association of Cancer Registry were followed for the overall registration process including data quality control. Ethical approval was taken from the Ethical Review Board of the NHRC.

**Result:**

Age-adjusted incidence (AAR) and mortality rates in Nepal were found 65.6 and 29 per 100,000 people, respectively. Every 1 in 14 men and 1 in 13 women were at risk of getting, and 1 in 28 men and 1 in 33 women were dying of cancer before age 75 in Nepal. The highest risk was found for lung cancer (1 in 80) followed by stomach and mouth among men, and in women, breast cancer (1 in 76) was the commonest among all followed by lung and cervix.

**Conclusion:**

Cancer has been growing as one of the major public health burdens in Nepal. Screening with cost-effective technology, awareness, and vaccination against HPV should be a government priority including revision of treatment protocols for cancers that have higher mortality to prevent further preventable life loss from malignancies.

## 1. Background

Cancer is one of the growing public health problems globally. According to GLOBOCAN (2020), there were 19.3 million new and 10 million cancer deaths in the global community, and the burden is expected to be raised every year [1]. LMICs have occupied a major share of global cancer as well as three-quarters of all cancer deaths [2, 3]. There has been an increasing burden of cancer for more than a decade in Nepal. More than twenty-two thousand (22008) new cases and fourteen thousand (14,704) deaths in Nepal were estimated in 2022 [11], and the burden has almost doubled since 1990 [4, 5]. Nepal Burden of Disease 2019 (NBoD) reported cancer to be the third most common cause of death [5]. Despite the increasing burden, a systematic national cancer control strategy and prevention program to fight against cancer has not yet been formulated in Nepal.

PBCR is the ultimate source for developing evidence-based cancer prevention and control policy and guiding tools for well-organized investment as well as to evaluate the effectiveness of the interventions at the population level [6].

NHRC has started PBCR to provide correct and comprehensive cancer data since 1^st^ January 2018. The PBCR included the districts from urban, suburban, and rural regions. The present study is aimed at describing the overall cancer status of the country including the cumulative risk of having cancer before the age of 75 years and mortality-to-incidence ratio of each cancer based on registry data which speak out the real burden of the disease.

## 2. Methods and Materials

The data was collected by PBCR Nepal operated by NHRC in 9 out of 77 districts. The districts were selected from three geographical regions of the country (Kathmandu, Lalitpur, and Bhaktapur from the urban region; Siraha, Saptari, Dhanusha, and Mahottari (SSDM) from the suburban region; and Rukum East and Rukum West from rural region). Urban, suburban, and rural were categorized based on facilities like medical, education, and transportation available in the region. The registry covered 21% of the estimated total population of the country which was more than six million (6,249,769). Geographically, 3,151,205 population from urban, 2,872,760 suburban, and 225,805 rural regions were covered by the registry. The estimated population was calculated using an inter-census method based on the census population of 2001 and 2011 [23]. Relevant data on cancer incidence and mortality was collected using direct and indirect methods through health facility-based and community-based approaches. In the health facility-based approach, trained data enumerators reviewed relevant medical documents including histopathology reports, radio-diagnostic reports, and treatment summaries of death certificates from medical records of hospitals, laboratories, and hospices to extract necessary information on cancer incidence and mortality. In a community-based approach, primary data was collected via face-to-face interviews with the patient and/or their family members. The verbal autopsy method was used in those cases whose clinical reports were burnt after death. Trained registry personnel checked and verified each dataset for completeness and correctness. We used CanReg5 software to check duplication and data entry. Detailed methodology of data collection can also be found in the previously published reports and journal articles [7–9]. Ethical Review Board (ERB) of NHRC has granted ethical approval for this study. Quality control parameters such as the proportion of cases registered with death certificate only (DCO), cases with primary site unknown (PSU), mortality-to-incidence ratio (M : I), and the proportion of cases with microscopic verification (MV) were assessed.

For analysis purposes, we have used incidence and mortality cases registered in 2019 and the estimated population of specified regions of the same year including the world standard population to standardize the rate. Age-adjusted rates were calculated by multiplying age-specific rates by the world standard population for each age group and site. Estimation of lifetime risk of developing cancer before 75 years old was calculated using the standard formula “{5 × (summation of the age − specific rate of 0 − 74 years)}/100,000” for cumulative rate and “1 − exp (−cumulative rate)” for cumulative risk [6].

## 3. Result/Findings

Overall, 3295 new cancer cases and 1427 deaths were registered in nine districts including urban, suburban, and rural regions of the country. Among the incidence cases, 1559 (47.3%) were men (S1_Table), and 1736 (52.7%) were women (S2_Table). Out of 1,427 deaths, 759 (53.2%) were men (S3_Table), and 668 (46.8%) were women (S4_Table).

Concerning quality control parameters, 76.4% of registered cases (91.7% in urban, 51.6% in suburban, and 60.2% in rural) were microscopically verified. Similarly, 1.1% of cases were registered based on DCO, and 2.8% had primary site unknown (S6_Table).

### 3.1. Incidence and Mortality Rate

Overall age-standardized (world) incidence (AAR) and mortality rates per 100,000 population were 65.6 and 29, respectively. In men, the cancer incidence and mortality rates (AAR) were 63.5 and 31.2 per 100,000, respectively. Similarly, in women, the rate was 68.1 and 27 per 100,000, respectively. The incidence and mortality rates were found to vary in each geographical region. The urban region has the highest incidence and mortality in both men and women. The overall mortality-to-incidence ratio (MIR) was found 0.43 (0.49 for men and 0.23 for women).

While looking for ethnic diversities, cancer was more common among *Brahmin/Chhetri* caste (30.8%), followed by *Newar* (22.7%), *Janajati* (19.7%), *Terai* caste (16%), *Dalit* (4.8%), *Muslim* (2.5%), and others (caste unknown) (3.6%), respectively.

## 4. Cumulative Risk of Cancer Incidence and Mortality before Age 75 Years

Overall, every 1 in 14 men and 1 in 13 women in Nepal were at risk for developing cancer before age 75. The risk was highest in the urban region for both men and women. Similarly, more men than women have died of cancer before 75 years of age in Nepal. The risk of dying due to cancer was lower in the suburban region compared to urban and rural regions (Tables 1 and 2).

## 5. Common Cancer Pattern

Our finding revealed that the commonest cancers in Nepal (among both sexes) were lung (12.3%) followed by breast (10.2%), gallbladder (6.6%), stomach (5.9%), cervix (5.8%), mouth (3.7%), colon (3.5%), liver (3.2%), ovary (3%), and thyroid (3%), respectively.

**Figure 1:**
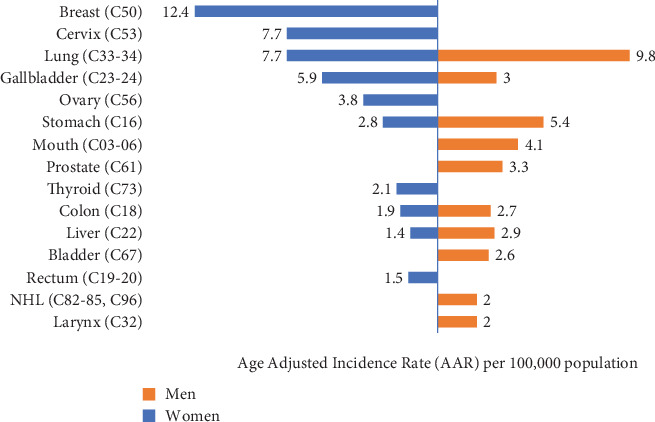
The overall patterns of leading cancer sites in urban, suburban, and rural regions by sex.

Among men, the leading cancer was lung followed by stomach, mouth, prostate, and gallbladder, respectively. Likewise in women, the breast was the most common cancer site followed by the cervix, lung, gallbladder, and ovary, respectively. Although the pattern of cancer found varies in urban, suburban, and rural regions (S5_Table), the overall pattern of cancer incidence in men and women is shown in Figure 1.

## 6. Cancer Risk before Age 75 Years

We found that men were at the greatest risk of getting lung cancer in Nepal before they reached 75 years old. They had a higher risk of developing stomach cancer followed by mouth and prostate cancer. Similarly, women were at the highest risk of being suffered from breast cancer followed by lung and cervix cancer. Moreover, cancer of the lung, gallbladder, and liver had a higher mortality-to-incidence ratio (MIR) in men, whereas, in women, lung followed by colon and gall bladder cancer had a higher MIR. The details have been elaborated in Table 3.

## 7. The Most Lethal Cancers in Nepal

Out of the total registered death cases in 2019, the most fatal cancers among both sexes were lung (20.8%), gallbladder (10%), stomach (6%), breast (5.9%), and liver (5.3%), respectively.

Among men, cancer of the lung covered the highest proportion of all deaths followed by gallbladder, stomach, liver, and mouth, respectively. Almost more than half (51.2%) of cancer deaths were by the top five cancers in men.

**Figure 2:**
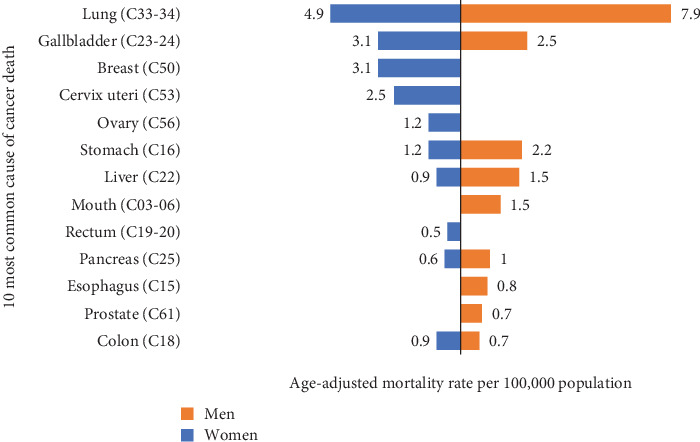
The top 10 fatal cancers of urban, suburban, and rural regions by sex.

Similarly, lung followed by gallbladder cancer was the most common cause of death in women as well. Breast cancer was the third most fatal cancer in Nepalese women. The five most common cancers comprised more than half (55.6%) of total cancer deaths among women which is shown in Figure 2.

## 8. Discussion

Overall cancer incidence and mortality were found to be significantly lower compared to the latest estimates of GLOBOCAN, which reported 80.9 and 54.8 per 100,000 incidence and mortality, respectively, and the Global Burden of Disease, which reported 101.8 and 86.5 per 100,000, respectively [4, 11]. However, the rates varied notably among the two global databases.

Cancer incidence and mortality rates found vary across the different geographical regions of the country. Urban region has higher rates compared to suburban and rural regions. Awareness, education, and plenty of medical and diagnostic facilities in the urban region promote health-seeking behaviors and increased diagnosis. In addition, a higher prevalence of risk factors such as pollution, urban lifestyles (less physical activity), and consumption of processed foods increased the risk of cancer in urban regions [9].

Cancer burden in women versus men (1 : 0.9) was higher for a long time, which was comparable to the other studies [8, 12, 13]. Similar findings were found in the countries bordered by Nepal [15, 16]. In contrast to incidence, cancer mortality was found to be higher in men versus women (1.1 : 1), according to the similar findings reported by PBCR Nepal 2018 [8] and previously published journals as well [4].

Cancer was found common among the ethnic group “*Brahmin/Chhetri*” in comparison to other groups in Nepal. The *Brahmin/Chhetri* people in Nepal occupy a higher proportion (27.7%) of the population, and they are relatively sound in terms of economy and education than other castes such as *Dalit*, *Muslim*, *Janajati*, and other *Terai* castes [16, 17]. Scholars reported that financial hardship and a low level of education delayed seeking diagnostic intervention resulting decreased incidence rate. Thus, poverty and poor awareness might decrease health-seeking practices among Dalit, Muslim, Janajati, and other Tarai castes, resulting in a low incidence [18].

Lung cancer in Nepal was seen as the commonest and the most lethal cancer [4, 8, 12]. Overall mortality of lung cancer (20.8%) was almost twofold higher as compared to incidence (12.3%). The cumulative risk of getting lung cancer before age 75 was found comparable to GLOBOCAN estimates (1.29%) for Nepal. However, the recent estimation of the proportion of lung cancer in India (10.6% men and 3.7% women) was lower compared to Nepal [11, 34]. The women residing in rural regions suffered more from lung cancer (22.2%) compared to women from urban (11.3%) and suburban regions (8.1%). Factors such as exposure to pollution (indoor and outdoor) and tobacco smoking are responsible for the development of lung cancer [19–21]. More than a quarter (28.9%) of people were current tobacco users in Nepal; among them, 48.3% were men, and 11.6% were women [22]. In addition, cities in Nepal have become more polluted due to uncoordinated development projects, rapid industrialization, and urbanization. On the other hand, overall 74% (86% in rural and 14% in urban) households used solid fuels (firewood and cow briquette) for cooking, and they (especially women) were exposed to indoor smoke at least 6 hours a day [23]. Consequently, long-term exposure to known human carcinogens in the smoke and dust particles increases the risk of lung cancer [24–27].

Our findings revealed that breast cancer surpassed cervix cancer. Breast cancer was estimated as the leading cancer (19.1%) and third most common cause of cancer death (12.3%) among women. Geographically, breast cancer incidence was higher among women in urban (21%) than in suburban (16.9%) and rural regions (15.6%). Similar findings have been witnessed in PBCR 2018 [8]. Although breast cancer was the most common cancer among women in Nepal, the age-adjusted rate of it was much higher in China (AAR 37.7 per 100,000) [28] and India (25.8 per 100,000) compared to Nepal [29]. Scholars suggested that factors such as early menarche (before the age of 12), late menopause (after 55 years of age), null parity or first child after 30, alcohol use, decreased duration of breastfeeding, urban residency, high BMI (greater than 20-25 kg/m^2^), less physical activity, consumption of a diet with high red meat, and tobacco use were significantly associated with risk of breast cancer [29, 30]; however, breast cancer in Nepalese women was found associated with late menarche (greater than 14 years), early first full-term pregnancy (before 40 weeks), and longer duration of breastfeeding. Besides, high dietary fat, excessive alcohol consumption, smoking, women in hormone replacement therapy, and exposure to radiation were associated with increased risk of breast cancer in Nepal [30].

The cervix cancer was estimated to be the second most common cancer among women which covered 11.1% of cases in Nepal. Women in suburban (14.6%) and rural regions (13.3%) suffered more from cervix cancer compared to urban women (9.1%). In contrast to current findings, the estimated proportion of cervix cancer 19.4% in Nepal by the GLOBOCAN was almost doubled [11]. Our finding reported that cervix cancer is responsible for 9.1% of cancer deaths among women. In Nepal, more than a quarter (28.8%) of death were from women's cancers (breast, cervix, corpus uteri, vagina, ovary, and other genital organs) which was significantly higher compared to 17.6% of deaths from the same cause in India [15]. The major known risk factor for cancer of the cervix, vagina, and vulva is human papilloma virus (HPV) which can be prevented by vaccination. There has not been access to nationwide HPV vaccination in Nepal; however, the government of Nepal issued “The National Guidelines for Cervical Cancer Screening and Prevention (CCSP)” in 2010 to screen at least half (up to 70% revised in 2017) of women aged 30-60 years which emphasized cervix cancer screening through VIA as one of the priorities programs. Despite of accessibility of screening services at local level health facilities, screening utilization was far below the target level where just 8.2% of women were screened by 2019 [31]. Inadequate knowledge and understanding regarding the cause, risk factors, and prevention as well as embarrassment were the major barriers to low utilization of cervical screening services in Nepal [14]. The government should focus on media coverage regarding the accessibility of screening service facilities as well as the need for screening to promote optimal utilization of the services at the local level.

Our finding showed mouth cancer as the leading cancer (13%, AAR 6.2 per 100,00 population) among men in suburban regions. The first-year report of PBCR reported a similar incidence of mouth cancer in 2018 [32]. The finding was almost similar to mouth cancer incidence (AAR 6.7 per 100,000) in the Muzaffarpur district of Bihar province of India [33] which shared a border with the southern part of the suburban region (Siraha, Saptari, Dhanusha, and Mahottari districts) of Nepal. It is already known that tobacco use is responsible for causing most of the cancer of the oral cavity including the mouth, and tobacco control is the most effective way of cancer prevention.

Similarly, cancer of the stomach, prostate, and gall bladder were among the top cancers in Nepal which had relatively higher mortality in response to incidence. Mortality-to-incidence ratio reflects the intervention of cancer control programs and also provides population-based survival estimates for each cancer [10]. MIR can give evidence to stress the need for promoting screening services for the early detection of cancer as well as to improve treatment protocols for some cancers that have higher mortality.

The last but not the least, this study has some limitations. The mortality data might be under registration. Lack of information regarding the cause of death at the civil registration and vital statistics (CRVS), inadequate documentation in the hospital-based cancer registry (HBCR), and unavailability of an electronic medical record system in Nepal are the major hurdles for collecting mortality data. Besides, PBCR Nepal used every possible source of information to collect relevant data on incidence and mortality. Phone calls to each incidence case during residence confirmation and follow-up; reviewing clinical reports, summaries, and death certificates of those who died in health facilities; and verbal autopsy methods during community visits were the major approaches used in case finding for mortality data. Moreover, the proportion of MV in suburban and rural regions was considerably lower than in the urban registry, and this is a reflection of the inaccessibility of advanced diagnostic and treatment facilities in the region. To capture as many cases as possible, PBCR Nepal stressed data collection through community visits in the suburban and rural regions, and more than half (51.9%) of data from the suburban and 44.3% cases from rural were identified by data enumerators with the help of FCHVs, community leaders, community health workers, etc. [7]. In addition, we have followed the guidelines and principles of IACR [6] while going through various processes of data collection and registration help to ensure data quality control of this study.

## 9. Conclusion

Every 1 in 13 men and every 1 in 14 women had the possibility of having cancer before they reached 75 years in Nepal. As more than half of cancer deaths were covered by the top five cancers in both men and women, concerned authorities should focus provision of screening services and their proper utilization to promote early detection of cancers with cost-effective technology including awareness of major risk factors as well as vaccination against HPV. The Government of Nepal should prioritize effective cancer prevention and control interventions through the national cancer control and prevention strategy which is not being formulated yet. The role of Civil Registration and Vital Statistics (CRVS) should be expanded along with the provision of cause of death for robust mortality data. Population-based cancer registry should be conducted in all provinces to guide strategies and evaluate intervention. The findings will be a key resource for policymakers, researchers, public health professionals, and clinicians making evidence-based decisions in the field of national cancer control programs.

## Figures and Tables

**Table 1:** Age-adjusted incidence (AAR) rate (0-75+)) and estimation of risk before age 75 in different geographical regions.

Registry site	AAR		Cum risk %		Possibility of the number of people likely to develop cancer before age 75	
	Men	Women	Men	Women	Men	Women
Urban	86.7	90.8	10.26	10.17	1 in 10	1 in 10
Suburban	47.8	49	4.87	5.44	1 in 21	1 in 18
Rural	52.5	47.3	5.72	4.72	1 in 17	1 in 21
All regions	63.5	68.1	7.05	7.51	1 in 14	1 in 13

**Table 2:** Age-adjusted mortality rate (0-75+) and estimation of risk for cancer death before age 75 years in different geographical regions.

Registry site	AAR		Cum risk %		Possibility of the number of people likely to die due to cancer before age 75	
	Men	Women	Men	Women	Men	Women
Urban	42.2	34.3	5.23	4.06	1 in 19	1 in 25
Suburban	24.0	19.9	2.52	2.24	1 in 40	1 in 47
Rural	27.6	25.5	2.88	2.86	1 in 35	1 in 35
All regions	31.2	27.0	3.60	3.02	1 in 28	1 in 33

**Table 3:** Cumulative risk of 10 most common cancers (0-74 years) and mortality-to-incidence ratio (MIR) among men and women in Nepal.

Cumulative risk of 10 most common cancers (0-74 years) and mortality-to-incidence ratio (MIR)							
Men				Women			
Common Cancer	Cum risk %	1 in number of men likely to have cancer	MIR	Common cancer	Cum risk %	1 in the number of women likely to have cancer	MIR
Lung (C33-34)	1.26	80	0.81	Breast (C50)	1.31	76	0.13
Stomach (C16)	0.65	153	0.45	Lung (C33-34)	0.94	107	0.50
Mouth (C03-06)	0.46	216	0.36	Cervix uteri (C53)	0.90	111	0.13
Prostate (C61)	0.35	283	0.24	Gallbladder (23-24)	0.75	133	0.33
Gallbladder (C23-24)	0.35	288	0.80	Ovary (56)	0.39	255	0.24
Liver (C22)	0.33	303	0.78	Stomach (C16)	0.37	271	0.27
Colon (C18)	0.32	311	0.30	Colon (C18)	0.24	414	0.45
Bladder (C67)	0.27	368	0.23	Thyroid (C73)	0.19	514	0.03
Larynx (C32)	0.25	397	0.38	Liver (C22)	0.19	528	0.15
NHL (C82-85, 96)	0.24	414	0.25	Tongue (C01-02)	0.16	614	0.17

## Data Availability

All relevant data are available within the manuscript and in supplementary files to support the findings of this study.

## Supplementary Materials

Supplementary 1 S1_Table: cancer incidence among men.

Supplementary 2 S2_Table: cancer incidence among women.

Supplementary 3 S3_Table: cancer mortality among men.

Supplementary 4 S4_Table: cancer mortality among women.

Supplementary 5 S5_Table: five most common cancers in urban, suburban, and rural regions of the country.

Supplementary 6 S6_Table: quality indicators of cases registered in urban, suburban, and rural regions.
