# Supplementary material for: Cancer Risk in Nepal: An Analysis from Population-Based Cancer Registry of Urban, Suburban, and Rural Regions
Source: J Cancer Epidemiol. 2024 Jul 10;2024:4687221. doi: 10.1155/2024/4687221 (PMC11949594; doi:10.1155/2024/4687221)
Supplement: Supplementary 1 — S1_Table: cancer incidence among men. [file 4687221.f1.docx]

**Cancer Risk in Nepal: An Analysis from Population-Based Cancer Registry of Urban, Sub-urban and Rural Regions**

Corresponding Author:

Uma Kafle Dahal (dahaluma1@gmail.com)

Gehanath Baral (baraldr@gmail.com)

Supplementary Table 1 (S1_Table)

This is the standard registry table based on the 2019 data created by the author/s

| **S1_Table: Estimation of Cancer Incidence Cases, Age Specific Rate, Age Standardized (world) Rate, Crude Rate (CR), Truncated Rate (aged 35-65) and Cumulative Risk Percent (0-74) Among Men** | | | | | | | | | | | | | | | | | | | | | | | |
| --- | --- | --- | --- | --- | --- | --- | --- | --- | --- | --- | --- | --- | --- | --- | --- | --- | --- | --- | --- | --- | --- | --- | --- |
| **ICD (10th)** | **SITES** | **Total** |  | **Age-Group** | | | | | | | | | | | | | | | |  |  |  |  |
|  |  |  | **(%)** | **0-4** | **5-9** | **10-14** | **15-19** | **20-24** | **25-29** | **30-34** | **35-39** | **40-44** | **45-49** | **50-54** | **55-59** | **60-64** | **65-69** | **70-74** | **75+** | **CR** | **AAR** | **TR** | **Cum risk % (0-74)** |
| **C00** | Lip | 10 | 0.6 | - | - | - | - | - | - | - | - | - | 0.7 | 1.6 | 3.9 | - | 1.5 | 4.5 | - | 0.3 | 0.4 | 0.9 | 0.06 |
| **C01-02** | Tongue | 35 | 2.2 | - | - | - | - | - | 0.8 | 0.9 | 0.5 | 1.6 | 2.0 | 3.2 | 5.9 | 6.7 | 4.6 | 4.5 | 6.9 | 1.1 | 1.4 | 2.9 | 0.15 |
| **C03-06** | Mouth | 101 | 6.5 | - | - | - | - | - | 0.4 | 1.4 | 3.3 | 4.8 | 4.7 | 12.1 | 17.7 | 13.4 | 16.9 | 18.1 | 23.0 | 3.2 | 4.1 | 8.5 | 0.46 |
| **C07-08** | Salivary glands | 7 | 0.4 | - | - | - | - | - | - | - | - | - | 0.7 | - | - | 1.1 | 4.6 | - | 4.6 | 0.2 | 0.3 | 0.3 | 0.03 |
| **C09** | Tonsil | 5 | 0.3 | - | - | - | - | - | - | - | 0.5 | - | 1.3 | - | 1.0 | 1.1 | - | - | 0.0 | 0.2 | 0.2 | 0.6 | 0.02 |
| **C10** | Other oropharynx | 7 | 0.4 | - | - | - | - | - | - | - | - | - | 0.7 | 0.8 | 1.0 | 1.1 | - | 2.3 | 4.6 | 0.2 | 0.3 | 0.5 | 0.03 |
| **C11** | Nasopharynx | 9 | 0.6 | - | - | 0.3 | - | - | - | - | 0.5 | 0.5 | 0.7 | 0.8 | 1.0 | 2.2 | - | - | 2.3 | 0.3 | 0.3 | 0.9 | 0.03 |
| **C12-13** | Hypopharynx | 17 | 1.1 | - | - | - | - | - | - | - | - | - | - | 2.4 | 5.9 | 2.2 | 3.1 | 6.8 | 2.3 | 0.5 | 0.7 | 1.4 | 0.10 |
| **C14** | Pharynx unspecified | 6 | 0.4 | - | - | - | - | - | 0.4 | 0.5 | - | - | - | - | - | 1.1 | 1.5 | 4.5 | - | 0.2 | 0.2 | 0.1 | 0.04 |
| **C15** | Oesophagus | 40 | 2.6 | - | - | - | - | - | - | - | - | 2.1 | 2.7 | 1.6 | 3.9 | 6.7 | 10.8 | 9.0 | 20.7 | 1.3 | 1.7 | 2.6 | 0.18 |
| **C16** | Stomach | 128 | 8.2 | - | - | - | - | 1.3 | 0.8 | 0.9 | 1.9 | 1.1 | 6.0 | 9.7 | 14.8 | 19.0 | 21.5 | 54.2 | 52.9 | 4.1 | 5.4 | 7.7 | 0.65 |
| **C17** | Small intestine | 3 | 0.2 | - | - | - | - | - | - | - | - | 0.5 | - | 0.8 | 1.0 | - | - | - | - | 0.1 | 0.1 | 0.4 | 0.01 |
| **C18** | Colon | 67 | 4.3 | - | - | - | - | 0.3 | 1.1 | 0.9 | 1.9 | 1.6 | 2.7 | 10.5 | 5.9 | 8.9 | 16.9 | 13.6 | 13.8 | 2.2 | 2.7 | 4.8 | 0.32 |
| **C19-20** | Rectum | 46 | 3.0 | - | - | - | 0.6 | 0.3 | 0.8 | 1.4 | 1.0 | 3.2 | 2.0 | 1.6 | 3.9 | 5.6 | 9.2 | 13.6 | 9.2 | 1.5 | 1.8 | 2.7 | 0.22 |
| **C21** | Anus | 3 | 0.2 | - | - | - | - | - | - | - | - | 0.5 | 0.7 | - | - | - | - | - | 2.3 | 0.1 | 0.1 | 0.2 | 0.01 |
| **C22** | Liver | 69 | 4.4 | - | - | 0.3 | 0.3 | - | - | 0.5 | 1.0 | 2.7 | 4.0 | 1.6 | 9.8 | 13.4 | 12.3 | 20.3 | 27.6 | 2.2 | 2.9 | 4.7 | 0.33 |
| **C23-24** | Gallbladder etc. | 74 | 4.7 | - | - | - | 0.3 | 0.6 | - | 0.5 | 1.0 | 3.2 | 2.0 | 8.1 | 10.8 | 8.9 | 18.4 | 15.8 | 25.3 | 2.4 | 3.0 | 5.0 | 0.35 |
| **C25** | Pancreas | 34 | 2.2 | - | - | - | 0.3 | - | - | 0.5 | - | 0.5 | 2.0 | 3.2 | 4.9 | 4.5 | 3.1 | 15.8 | 13.8 | 1.1 | 1.4 | 2.2 | 0.17 |
| **C30-31** | Nose, sinuses etc. | 7 | 0.4 | - | - | - | - | - | 0.4 | - | - | 1.6 | 1.3 | - | - | - | 1.5 | - | - | 0.2 | 0.3 | 0.6 | 0.02 |
| **C32** | Larynx | 47 | 3.0 | - | - | - | - | - | 0.4 | - | 1.0 | - | 0.7 | 3.2 | 3.9 | 5.6 | 15.4 | 20.3 | 25.3 | 1.5 | 2.0 | 2.1 | 0.25 |
| **C33-34** | Trachea, bronchus and lung | 227 | 14.6 | - | - | - | 0.3 | 0.6 | - | 1.4 | 1.4 | 1.1 | 2.7 | 10.5 | 34.5 | 35.8 | 67.6 | 97.2 | 103.5 | 7.3 | 9.8 | 11.8 | 1.26 |
| **C37-38** | Other thoracic organs | 3 | 0.2 | - | - | - | - | - | 0.4 | - | - | - | 0.7 | - | - | 1.1 | - | - | - | 0.1 | 0.1 | 0.3 | 0.01 |
| **C40-41** | Bone | 30 | 1.9 | - | 0.3 | 1.1 | 1.4 | 1.3 | 0.8 | 0.9 | 1.0 | 0.5 | 0.7 | - | 1.0 | 1.1 | 1.5 | 4.5 | 6.9 | 1.0 | 1.0 | 0.7 | 0.08 |
| **C43** | Melanoma of skin | 4 | 0.3 | - | - | - | - | - | - | - | - | - | - | - | 1.0 | - | 4.6 | - | - | 0.1 | 0.2 | 0.1 | 0.03 |
| **C44** | Other skin | 22 | 1.4 | - | - | 0.3 | - | - | - | - | - | 1.6 | 1.3 | 3.2 | 3.0 | 2.2 | - | 6.8 | 9.2 | 0.7 | 0.9 | 1.8 | 0.09 |
| **C45** | Mesothelioma | 4 | 0.3 | - | - | - | - | 0.3 | - | - | 0.5 | - | - | - | - | - | 1.5 | 2.3 | - | 0.1 | 0.1 | 0.1 | 0.02 |
| **C46** | Kaposi sarcoma | - | - | - | - | - | - | - | - | - | - | - | - | - | - | - | - | - | - | - | - | - | - |
| **C47,C49** | Connective and soft tissue | 14 | 0.9 | - | - | - | 0.3 | 1.0 | - | - | 0.5 | 1.6 | - | 1.6 | 2.0 | 1.1 | - | - | 2.3 | 0.5 | 0.5 | 1.1 | 0.04 |
| **C50** | Breast | 7 | 0.4 | - | - | - | - | - | - | - | - | - | 0.7 | 0.8 | - | - | 6.1 | - | 2.3 | 0.2 | 0.3 | 0.3 | 0.04 |
| **C60** | Penis | 20 | 1.3 | - | - | - | - | 0.3 | 0.4 | 0.5 | - | 0.5 | 1.3 | 1.6 | 2.0 | 2.2 | 3.1 | 9.0 | 4.6 | 0.6 | 0.8 | 1.2 | 0.10 |
| **C61** | Prostate | 72 | 4.6 | - | - | - | - | 0.3 | - | - | - | - | - | 0.8 | - | 13.4 | 24.6 | 31.6 | 64.4 | 2.3 | 3.3 | 1.9 | 0.35 |
| **C62** | Testis | 11 | 0.7 | - | - | - | - | 0.6 | 0.8 | 0.5 | 1.0 | 0.5 | 0.7 | - | 1.0 | - | - | - | 2.3 | 0.4 | 0.4 | 0.5 | 0.03 |
| **C63** | Other male genital organs | - | - | - | - | - | - | - | - | - | - | - | - | - | - | - | - | - | - | - | - | - | - |
| **C64** | Kidney | 29 | 1.9 | 1.2 | - | - | 0.3 | 0.3 | 0.4 | - | 0.5 | 1.1 | 3.3 | 2.4 | 3.0 | 6.7 | - | 4.5 | 2.3 | 0.9 | 1.2 | 2.6 | 0.12 |
| **C65** | Renal pelvis | - | - | - | - | - | - | - | - | - | - | - | - | - | - | - | - | - | - | - | - | - | - |
| **C66** | Ureter | 1 | 0.1 | - | - | - | - | - | - | - | - | - | - | - | - | - | 1.5 | - | - | - | - | - | 0.01 |
| **C67** | Bladder | 61 | 3.9 | - |  | - | - | - | - | 0.9 | 1.0 | 0.5 | 0.7 | 2.4 | 6.9 | 7.8 | 13.8 | 20.3 | 46.0 | 2.0 | 2.6 | 2.7 | 0.27 |
| **C68** | Other urinary organs | - | - | - | - | - | - | - | - | - | - | - | - | - | - | - | - | - | - | - | - | - | - |
| **C69** | Eye | 4 | 0.3 | 1.6 | - | - | - | - | - | - | - | - | - | - | - | - | - | - | - | 0.1 | 0.2 | - | 0.01 |
| **C70-72** | Brain, nervous system | 35 | 2.2 | 0.4 | - | - | 0.3 | 1.0 | - | 3.6 | 1.0 | 2.7 | 4.0 | - | 3.9 | 3.4 | 1.5 | 2.3 | - | 1.1 | 1.2 | 2.4 | 0.12 |
| **C73** | Thyroid | 32 | 2.1 | - | - | - | - | 0.3 | 1.9 | 0.9 | 1.4 | 1.6 | 2.0 | - | 3.0 | 5.6 | 4.6 | 6.8 | 2.3 | 1.0 | 1.2 | 2.1 | 0.14 |
| **C74** | Adrenal gland | - | - | - | - | - | - | - | - | - | - | - | - | - | - | - | - | - | - | - | - | - | - |
| **C75** | Other endocrine | - | - | - | - | - | - | - | - | - | - | - | - | - | - | - | - | - | - | - | - | - | - |
| **C81** | Hodgkin disease | 13 | 0.8 | - | 0.9 | - | 0.6 | - | 0.4 | 0.9 | - | 1.1 | - | 0.8 | - | 1.1 | 1.5 | - | - | 0.4 | 0.4 | 0.5 | 0.04 |
| **C82-85,C96** | Non-Hodgkin lymphoma | 51 | 3.3 | - | 0.6 | 0.5 | - | 0.6 | 0.4 | 0.9 | 0.5 | 1.1 | 3.3 | 3.2 | 5.9 | 10.1 | 7.7 | 13.6 | 9.2 | 1.6 | 2.0 | 3.5 | 0.24 |
| **C88** | Immune-proliferative diseases | - | - | - | - | - | - | - | - | - | - | - | - | - | - | - | - | - | - | - | - | - | - |
| **C90** | Multiple myeloma | 17 | 1.1 | - | - | - | - | 0.3 | 0.4 | - | - | - | 0.7 | 0.8 | 3.0 | - | 3.1 | 6.8 | 11.5 | 0.5 | 0.7 | 0.6 | 0.07 |
| **C91** | Lymphoid leukemia | 15 | 1.0 | - | 1.2 | - | 0.6 | - | - | 0.5 | 0.5 | - | 1.3 | - | 2.0 | 1.1 | - | 2.3 | 2.3 | 0.5 | 0.5 | 0.7 | 0.05 |
| **C92-94** | Myeloid leukemia | 32 | 2.1 | - | - | 0.5 | 0.3 | 0.6 | 0.8 | 1.4 | 0.5 | 2.7 | 2.0 | 2.4 | 1.0 | 1.1 | 6.1 | 2.3 | 6.9 | 1.0 | 1.1 | 1.7 | 0.11 |
| **C95** | Leukemia unspecified | 25 | 1.6 | 1.6 | 0.3 | 0.3 | 0.6 | 0.6 | 0.8 | 1.8 | 1.0 | - | 2.0 | - | 1.0 | - | 3.1 | 2.3 | - | 0.8 | 0.9 | 0.7 | 0.08 |
| Myelo-proliferative disorders | | - | - | - | - | - | - | - | - | - | - | - | - | - | - | - | - | - | - | - | - | - | - |
| Myelo-dysplastic syndromes | | - | - | - | - | - | - | - | - | - | - | - | - | - | - | - | - | - | - | - | - | - | - |
| Other and unspecified* | | 115 | 7.4 | 0.4 | 0.6 | 0.8 | 1.1 | 0.6 | 1.5 | 1.4 | 2.9 | 3.8 | 6.0 | 8.1 | 13.8 | 15.6 | 21.5 | 29.4 | 20.7 | 3.7 | 4.5 | 7.5 | 0.54 |
| **Total** | | **1559** | **100.0** | **5.3** | **4.0** | **4.0** | **7.0** | **11.6** | **13.7** | **22.8** | **25.8** | **44.5** | **68.1** | **100.0** | **183.1** | **211.2** | **314.9** | **445.3** | **531.4** | **50.1** | **63.5** | **93.8** | **7.05** |

*Other and unspecified sites include ICD codes: C26, C48, C76, C77 and C80*
