# Supplementary material for: Cancer Risk in Nepal: An Analysis from Population-Based Cancer Registry of Urban, Suburban, and Rural Regions
Source: J Cancer Epidemiol. 2024 Jul 10;2024:4687221. doi: 10.1155/2024/4687221 (PMC11949594; doi:10.1155/2024/4687221)
Supplement: Supplementary 2 — S2_Table: cancer incidence among women. [file 4687221.f2.docx]

**Cancer Risk in Nepal: An Analysis from Population-Based Cancer Registry of Urban, Sub-urban and Rural Regions**

Corresponding Author:

Uma Kafle Dahal (dahaluma1@gmail.com)

Gehanath Baral (baraldr@gmail.com)

Supplementary Table (S2-Table)

This is the standard registry table based on the 2019 data created by the author/s

**S2_Table: Estimation of Cancer Incidence Cases, Age Specific Rate, Age Standardized (world) Rate, Crude Incidence Rate (CR), Truncated Rate (aged 35-65) and Cumulative Risk Percent (0-74) Among Women**

| **ICD (10th)** | SITE | **Total** | **RP (%)** | **Age-Group** | | | | | | | | | | | | | | | | **CR** | **AAR** | **TR** | **Cum Risk % (0-74)** |
| --- | --- | --- | --- | --- | --- | --- | --- | --- | --- | --- | --- | --- | --- | --- | --- | --- | --- | --- | --- | --- | --- | --- | --- |
|  |  |  |  | **0-4** | **5-9** | **10-14** | **15-19** | **20-24** | **25-29** | **30-34** | **35-39** | **40-44** | **45-49** | **50-54** | **55-59** | **60-64** | **65-69** | **70-74** | **75+** |  |  |  |  |
| **C00** | Lip | 3 | 0.2 | - | - | - | - | - | - | - | - | - | 0.7 | - | - | - | - | 2.3 | 2.0 | 0.1 | 0.1 | 0.1 | 0.01 |
| **C01-02** | Tongue | 29 | 1.7 | - | - | - | 0.3 | - | - | 0.4 | - | - | 2.0 | 0.9 | 5.1 | 6.7 | 8.0 | 9.2 | 6.0 | 0.9 | 1.2 | 2.1 | 0.16 |
| **C03-06** | Mouth | 21 | 1.2 | - | - | - | - | 0.3 | - | - | 0.4 | - | 2.0 | 2.6 | 1.0 | 5.6 | 1.6 | 6.9 | 6.0 | 0.7 | 0.9 | 1.7 | 0.10 |
| **C07-08** | Salivary glands | 5 | 0.3 | - | - | - | - | - | - | - | - | 0.5 | 1.4 | - | - | - | 1.6 | 2.3 | - | 0.2 | 0.2 | 0.4 | 0.03 |
| **C09** | Tonsil | 2 | 0.1 | - | - | - | - | - | - | - | - | - | - | - | - | - | 1.6 | - | 2.0 | 0.1 | 0.1 | - | 0.01 |
| **C10** | Other oropharynx | 4 | 0.2 | - | - | - | - | - | - | - | - | 0.5 | - | - | 1.0 | 1.1 | - | 2.3 | - | 0.1 | 0.2 | 0.4 | 0.02 |
| **C11** | Nasopharynx | 2 | 0.1 | - | - | - | - | - | - | - | - | - | 0.7 | - | - | - | 1.6 | - | - | 0.1 | 0.1 | 0.1 | 0.01 |
| **C12-13** | Hypopharynx | 4 | 0.2 | - | - | - | - | - | - | - | 0.4 | - | - | - | - | - | 3.2 | - | 2.0 | 0.1 | 0.2 | 0.1 | 0.02 |
| **C14** | Pharynx unspecified | 2 | 0.1 | - | - | - | - | - | - | - | - | - | - | - | - | 1.1 | - | - | 2.0 | 0.1 | 0.1 | 0.1 | 0.01 |
| **C15** | Oesophagus | 24 | 1.4 | - | - | - | - | - | - | 0.4 | 0.4 | - | - | 1.7 | 4.1 | 4.5 | 6.4 | 6.9 | 10.0 | 0.8 | 1.0 | 1.5 | 0.12 |
| **C16** | Stomach | 67 | 3.9 | - | - | - | - | - | - | 0.4 | 2.1 | 1.6 | 4.8 | 4.3 | 7.2 | 18.9 | 20.7 | 13.9 | 6.0 | 2.1 | 2.8 | 5.7 | 0.37 |
| **C17** | Small intestine | 4 | 0.2 | - | - | - | - | 0.3 | - | - | - | - | - | - | - | 1.1 | - | 2.3 | 2.0 | 0.1 | 0.2 | 0.1 | 0.02 |
| **C18** | Colon | 49 | 2.8 | - | - | - | - | 0.3 | 0.6 | 1.5 | 0.8 | 2.2 | 1.4 | 4.3 | 2.1 | 5.6 | 11.1 | 18.5 | 14.1 | 1.6 | 1.9 | 2.5 | 0.24 |
| **C19-20** | Rectum | 40 | 2.3 | - | - | - | - | 0.6 | 1.0 | 0.4 | 0.8 | 4.3 | 2.0 | 0.9 | 6.2 | 6.7 | 6.4 | 2.3 | 6.0 | 1.3 | 1.5 | 3.2 | 0.16 |
| **C21** | Anus | 3 | 0.2 | - | - | - | - | - | - | 0.4 | - | - | - | - | - | 1.1 | - | - | 2.0 | 0.1 | 0.1 | 0.1 | 0.01 |
| **C22** | Liver | 34 | 2.0 | - | - | - | - | - | - | - | - | 1.6 | 1.4 | - | 12.4 | 5.6 | 3.2 | 13.9 | 8.0 | 1.1 | 1.4 | 2.9 | 0.19 |
| **C23-24** | Gallbladder etc. | 144 | 8.3 | - | - | - | - | - | - | 3.0 | 4.2 | 4.9 | 6.8 | 11.2 | 28.8 | 24.5 | 39.8 | 27.7 | 14.1 | 4.6 | 5.9 | 11.8 | 0.75 |
| **C25** | Pancreas | 28 | 1.6 | - | - | 0.5 | - | - | 0.3 | - | 0.4 | 0.5 | 0.7 | 0.9 | 7.2 | 3.3 | 6.4 | 4.6 | 10.0 | 0.9 | 1.1 | 1.8 | 0.12 |
| **C30-31** | Nose, sinuses etc. | 2 | 0.1 | - | - | - | 0.3 | - | - | - | - | - | - | - | - | 1.1 | - | - | - | 0.1 | 0.1 | 0.1 | 0.01 |
| **C32** | Larynx | 17 | 1.0 | - | - | - | - | - | - | - | - | - | 1.4 | - | 5.1 | 6.7 | 1.6 | 2.3 | 4.0 | 0.5 | 0.7 | 1.8 | 0.09 |
| **C33-34** | Trachea, bronchus and lung | 181 | 10.4 | - | - | - | - | 0.3 | 0.6 | 0.8 | 1.7 | 2.2 | 6.1 | 10.4 | 22.7 | 35.6 | 43.0 | 64.7 | 76.3 | 5.8 | 7.7 | 11.1 | 0.94 |
| **C37-38** | Other thoracic organs | - | - | - | - | - | - | - | - | - | - | - | - | - | - | - | - | - | - | - | - | - | 0.00 |
| **C40-41** | Bone | 18 | 1.0 | 0.4 | - | 1.1 | 0.6 | 0.9 | - | 0.4 | - | - | 1.4 | 1.7 | 1.0 | - | - | 2.3 | 2.0 | 0.6 | 0.6 | 0.7 | 0.05 |
| **C43** | Melanoma of skin | 4 | 0.2 | - | - | - | - | - | - | - | - | 0.5 | 0.7 | - | 1.0 | 1.1 | - | - | - | 0.1 | 0.2 | 0.5 | 0.02 |
| **C44** | Other skin | 17 | 1.0 | - | - | - | - | - | - | - | 0.8 | 1.1 | - | 0.9 | 1.0 | 2.2 | 1.6 | 2.3 | 12.0 | 0.5 | 0.6 | 0.9 | 0.05 |
| **C45** | Mesothelioma | 1 | 0.1 | - | - | - | - | - | - | - | - | - | - | - | - | - | 1.6 | - | - | - | - | - | 0.01 |
| **C46** | Kaposi sarcoma | - | - | - | - | - | - | - | - | - | - | - | - | - | - | - | 0.0 | - | - | - | - | - | 0.00 |
| **C47,C49** | Connective and soft tissue | 10 | 0.6 | 0.4 | 0.3 | - | 0.3 | - | - | - | - | 1.6 | - | 0.9 | - | 1.1 | 1.6 | 2.3 | - | 0.3 | 0.4 | 0.6 | 0.04 |
| **C50** | Breast | 331 | 19.1 | - | - | - | - | 0.3 | 3.8 | 8.3 | 14.0 | 26.5 | 34.7 | 41.4 | 48.4 | 30.0 | 27.0 | 30.0 | 22.1 | 10.5 | 12.4 | 31.4 | 1.31 |
| **C51** | Vulva | 7 | 0.4 | - | - | - | - | - | - | - | - | 0.5 | - | 0.9 | - | 1.1 | - | 6.9 | 2.0 | 0.2 | 0.3 | 0.4 | 0.05 |
| **C52** | Vagina | 11 | 0.6 | - | - | - | - | - | - | - | 0.4 | 0.5 | 2.0 | 1.7 | 2.1 | 1.1 | - | - | 2.0 | 0.4 | 0.4 | 1.3 | 0.04 |
| **C53** | Cervix uteri | 193 | 11.1 | - | - | - | - | - | - | 1.9 | 6.8 | 12.4 | 19.7 | 24.2 | 16.5 | 28.9 | 33.4 | 37.0 | 22.1 | 6.1 | 7.7 | 17.3 | 0.90 |
| **C54** | Corpus uteri | 31 | 1.8 | - | - | - | - | - | - | 0.4 | 2.1 | - | 1.4 | 6.9 | 4.1 | 5.6 | 9.5 | - | - | 1.0 | 1.3 | 3.0 | 0.15 |
| **C55** | Uterus unspecified | 19 | 1.1 | - | - | - | - | - | - | - | 1.7 | 2.2 | 2.7 | 1.7 | 3.1 | 1.1 | 1.6 | 2.3 | - | 0.6 | 0.7 | 2.1 | 0.08 |
| **C56** | Ovary | 100 | 5.8 | - | 0.3 | - | 1.6 | 0.3 | 1.0 | 2.6 | 3.4 | 4.9 | 10.2 | 13.8 | 7.2 | 12.2 | 4.8 | 16.2 | 14.1 | 3.2 | 3.8 | 8.3 | 0.39 |
| **C57** | Other female genital organs | 1 | 0.1 | - | - | - | - | - | - | - | - | - | - | - | 1.0 | - | - | - | - | - | - | 0.1 | 0.01 |
| **C58** | Placenta | 3 | 0.2 | - | - | - | - | 0.3 | - | 0.4 | - | 0.5 | - | - | - | - | - | - | - | 0.1 | 0.1 | 0.1 | 0.01 |
| **C64** | Kidney | 13 | 0.7 | 0.4 | - | - | - | - | - | - | - | - | 1.4 | 2.6 | - | 1.1 | 3.2 | 6.9 | 2.0 | 0.4 | 0.6 | 0.8 | 0.08 |
| **C65** | Renal pelvis | 3 | 0.2 | - | - | - | - | - | - | - | - | - | - | 1.7 | - | 1.1 | - | - | - | 0.1 | 0.1 | 0.4 | 0.01 |
| **C66** | Ureter | 1 | 0.1 | - | - | - | - | - | - | - | - | - | - | - | 1.0 | - | - | - | - | - | - | 0.1 | 0.01 |
| **C67** | Bladder | 15 | 0.9 | - | - | - | - | - | - | - | - | 0.5 | - | 3.5 | 2.1 | 4.5 | 3.2 | - | 4.0 | 0.5 | 0.6 | 1.5 | 0.07 |
| **C68** | Other urinary organs | - | - | - | - | - | - | - | - | - | - | - | - | - | - | - | - | - | - | - | - | - | 0.00 |
| **C69** | Eye | 6 | 0.3 | 0.9 | 1.0 | - | - | - | - | - | - | - | - | - | - | - | - | 2.3 | - | 0.2 | 0.3 | - | 0.02 |
| **C70-72** | Brain, nervous system | 25 | 1.4 | 0.4 | 0.7 | 0.3 | 0.6 | - | 0.3 | 0.4 | 1.3 | 1.6 | 2.0 | 2.6 | 1.0 | 2.2 | 1.6 | - | 2.0 | 0.8 | 0.9 | 1.8 | 0.08 |
| **C73** | Thyroid | 67 | 3.9 | - | - | - | - | 1.2 | 3.2 | 4.5 | 2.5 | 6.5 | 4.8 | 4.3 | 3.1 | 3.3 | 3.2 | 2.3 | 4.0 | 2.1 | 2.1 | 4.2 | 0.19 |
| **C74** | Adrenal gland | 2 | 0.1 | - | - | 0.3 | - | - | - | - | 0.4 | - | - | - | - | - | - | - | - | 0.1 | 0.1 | 0.1 | 0.00 |
| **C75** | Other endocrine | 1 | 0.1 | - | - | - | - | - | - | - | - | - | - | - | 1.0 | - | - | - | - | - | - | 0.1 | 0.01 |
| **C81** | Hodgkin disease | 5 | 0.3 | - | 0.3 | - | - | - | 0.3 | - | 0.4 | - | 0.7 | - | - | - | - | 2.3 | - | 0.2 | 0.2 | 0.2 | 0.02 |
| **C82-85,C96** | Non-Hodgkin lymphoma | 34 | 2.0 | - | - | - | - | 0.9 | - | 1.1 | 1.3 | 1.1 | 1.4 | 4.3 | 1.0 | 4.5 | 8.0 | 4.6 | 8.0 | 1.1 | 1.3 | 2.1 | 0.14 |
| **C88** | Immunoproliferative diseases | - | - | - | - | - | - | - | - | - | - | - | - | - | - | - | - | - | - | - | - | - | 0.00 |
| **C90** | Multiple myeloma | 17 | 1.0 | - | - | - | - | - | - | - | 0.8 | - | 1.4 | 1.7 | 2.1 | 2.2 | 1.6 | 4.6 | 8.0 | 0.5 | 0.7 | 1.3 | 0.07 |
| **C91** | Lymphoid leukaemia | 9 | 0.5 | - | 0.3 | 0.5 | 0.3 | 0.3 | - | 0.4 | 0.4 | - | 0.7 | - | - | - | - | - | 2.0 | 0.3 | 0.3 | 0.2 | 0.01 |
| **C92-94** | Myeloid leukaemia | 17 | 1.0 | - | 0.3 | - | 0.6 | 0.9 | - | 1.1 | 0.8 | 0.5 | 0.7 | - | - | - | 4.8 | 2.3 | - | 0.5 | 0.5 | 0.4 | 0.06 |
| **C95** | Leukaemia unspecified | 11 | 0.6 | - | 0.3 | 0.5 | 0.3 | - | - | 0.4 | - | - | - | 0.9 | 2.1 | - | 3.2 | - | 2.0 | 0.4 | 0.4 | 0.4 | 0.04 |
| Myeloproliferative disorders | | - | - | - | - | - | - | - | - | - | - | - | - | - | - | - | - | - | - | - | - | - | 0.00 |
| Myelodysplastic syndromes | | - | - | - | - | - | - | - | - | - | - | - | - | - | - | - | - | - | - | - | - | - | 0.00 |
| Other and unspecified | | 99 | 5.7 | 1.3 | 1.3 | 0.3 | - | 0.6 | 1.3 | 1.1 | 1.3 | 2.7 | 7.5 | 6.9 | 10.3 | 10.0 | 28.6 | 23.1 | 16.1 | 3.2 | 4.0 | 6.0 | 0.48 |
| **Total** |  | **1736** | **100.0** | **4.0** | **5.0** | **3.6** | **5.1** | **7.6** | **13.0** | **30.2** | **50.1** | **82.2** | **124.6** | **159.6** | **212.2** | **242.5** | **294.3** | **328.0** | **297.2** | **55.3** | **68.1** | **134.1** | **7.51** |

*Other and unspecified sites include ICD codes: C26, C48, C75, C76, C77 and C80*
