# Supplementary material for: Cancer Risk in Nepal: An Analysis from Population-Based Cancer Registry of Urban, Suburban, and Rural Regions
Source: J Cancer Epidemiol. 2024 Jul 10;2024:4687221. doi: 10.1155/2024/4687221 (PMC11949594; doi:10.1155/2024/4687221)
Supplement: Supplementary 3 — S3_Table: cancer mortality among men. [file 4687221.f3.docx]

**Cancer Risk in Nepal: An Analysis from Population-Based Cancer Registry of Urban, Sub-urban and Rural Regions**

Corresponding Author:

Uma Kafle Dahal (dahaluma1@gmail.com)

Gehanath Baral (baraldr@gmail.com)

Supplementary Table 3 (S3_Table)

This is the standard registry table based on the 2019 data created by the author/s

**S3_Table: Estimation of Cancer Mortality Cases, Relative Proportions (RP), Age Specific Rate, Crude Mortality Rate (CR) Age Standardized (world) Rate (AAR), Truncated Rate (aged 35-65), and Cumulative Risk Percent (0-74) Among Men**

| **ICD (10th)** | SITES | **Total** | **RP (%)** | **Age Group (years)** | | | | | | | | | | | | | | | | **CR** | **AAR** | **TR** | **Cum Risk % (0-74)** |
| --- | --- | --- | --- | --- | --- | --- | --- | --- | --- | --- | --- | --- | --- | --- | --- | --- | --- | --- | --- | --- | --- | --- | --- |
|  |  |  |  | **0-4** | **5-9** | **10-14** | **15-19** | **20-24** | **25-29** | **30-34** | **35-39** | **40-44** | **45-49** | **50-54** | **55-59** | **60-64** | **65-69** | **70-74** | **75+** |  |  |  |  |
| **C00** | Lip | 1 | 0.1 | - | - | - | - | - | - | - | - | - | - | - | - | - | - | 2.3 | - | - | - | - | 0.01 |
| **C01-02** | Tongue | 14 | 1.8 | - | - | - | - | 0.3 | - | 0.9 | 0.5 | 1.1 | - | 0.8 | - | 3.4 | 1.5 | 4.5 | 2.3 | 0.5 | 0.5 | 0.9 | 0.06 |
| **C03-06** | Mouth | 36 | 4.7 | 0.4 | - | - | - | - | - | 0.5 | 1.0 | 1.6 | 2.0 | 7.3 | 2.0 | 6.7 | 3.1 | 11.3 | 4.6 | 1.2 | 1.5 | 3.2 | 0.18 |
| **C07-08** | Salivary glands | 4 | 0.5 | - | - | - | - | - | - | - | - | - | - | - | 1.0 | - | 1.5 | 2.3 | 2.3 | 0.1 | 0.2 | 0.1 | 0.02 |
| **C09** | Tonsil | - | - | - | - | - | - | - | - | - | - | - | - | - | - | - | - | - | - | - | - | - | - |
| **C10** | Other oropharynx | 3 | 0.4 | - | - | - | - | - | - | - | - | 0.5 | - | - | 1.0 | 1.1 | - | - | - | 0.1 | 0.1 | 0.4 | 0.01 |
| **C11** | Nasopharynx | 1 | 0.1 | - | - | - | - | - | - | - | - | - | 0.7 | - | - | - | - | - | - | - | - | 0.1 | - |
| **C12-13** | Hypopharynx | 12 | 1.6 | - | - | - | - | - | - | - | - | - | 0.7 | 0.8 | 2.0 | 2.2 | 6.1 | - | 4.6 | 0.4 | 0.5 | 0.8 | 0.06 |
| **C14** | Pharynx unspecified | 6 | 0.8 | - | - | - | - | - | - | 0.5 | - | 0.5 | - | - | - | 1.1 | 4.6 | - | - | 0.2 | 0.2 | 0.2 | 0.03 |
| **C15** | Oesophagus | 24 | 3.2 | - | - | - | - | - | - | - | - | 1.1 | - | 4.0 | 2.0 | 4.5 | 6.1 | 6.8 | 9.2 | 0.8 | 1.0 | 1.7 | 0.12 |
| **C16** | Stomach | 57 | 7.5 | - | - | - | - | 0.6 | 0.4 | 1.4 | 1.0 | 0.5 | 5.3 | 5.6 | 6.9 | 1.1 | 15.4 | 13.6 | 20.7 | 1.8 | 2.3 | 3.3 | 0.26 |
| **C17** | Small intestine | 1 | 0.1 | - | - | - | - | - | - | - | 0.5 | - | - | - | - | - | - | - | - | - | - | 0.1 | - |
| **C18** | Colon | 20 | 2.6 | - | - | - | 0.3 | 0.3 | 0.4 | 0.9 | 0.5 | 1.6 | 1.3 | 1.6 | 1.0 | 1.1 | 1.5 | 6.8 | 2.3 | 0.6 | 0.7 | 1.2 | 0.09 |
| **C19-20** | Rectum | 9 | 1.2 | - | - | - | - | 0.3 | 0.4 | - | 0.5 | - | 1.3 | 0.8 | - | - | 1.5 | - | 4.6 | 0.3 | 0.3 | 0.5 | 0.02 |
| **C21** | Anus | 4 | 0.5 | - | - | - | - | - | - | 0.5 | - | 0.5 | - | 0.8 | - | - | - | - | 2.3 | 0.1 | 0.1 | 0.2 | 0.01 |
| **C22** | Liver | 54 | 7.1 | 0.4 | - | - | - | 0.3 | - | - | 1.9 | 2.1 | 4.0 | 0.8 | 5.9 | 7.8 | 7.7 | 22.6 | 20.7 | 1.7 | 2.2 | 3.5 | 0.27 |
| **C23-24** | Gallbladder etc. | 59 | 7.8 | - | - | - | - | - | - | - | 1.0 | 2.7 | 2.0 | 6.4 | 8.9 | 3.4 | 20.0 | 13.6 | 23.0 | 1.9 | 2.5 | 3.7 | 0.29 |
| **C25** | Pancreas | 26 | 3.4 | - | - | - | - | - | 0.4 | 0.5 | - | 0.5 | 0.7 | 2.4 | 4.9 | 2.2 | 3.1 | 11.3 | 11.5 | 0.8 | 1.1 | 1.5 | 0.13 |
| **C30-31** | Nose, sinuses etc. | 1 | 0.1 | - | - | - | - | - | 0.4 | - | - | - | - | - | - | - | - | - | - | - | - | - | - |
| **C32** | Larynx | 18 | 2.4 | - | - | - | - | - | - | - | - | - | 0.7 | 2.4 | 3.0 | 2.2 | 4.6 | - | 13.8 | 0.6 | 0.8 | 1.2 | 0.06 |
| **C33-34** | Trachea, bronchus and lung | 183 | 24.1 | - | - | - | 0.3 | - | - | 1.8 | 1.0 | 0.5 | 4.0 | 9.7 | 25.6 | 34.6 | 44.5 | 74.6 | 87.4 | 5.9 | 7.9 | 10.4 | 0.98 |
| **C37-38** | Other thoracic organs | - | - | - | - | - | - | - | - | - | - | - | - | - | - | - | - | - | - | - | - | - | - |
| **C40-41** | Bone | 10 | 1.3 | - | - | - | 0.6 | 0.6 | - | - | 0.5 | 0.5 | 0.7 | - | 1.0 | - | 1.5 | 2.3 | - | 0.3 | 0.3 | 0.5 | 0.04 |
| **C43** | Melanoma of skin | 1 | 0.1 | - | - | - | - | - | - | - | - | - | 0.7 | - | - | - | - | - | - | - | - | 0.1 | - |
| **C44** | Other skin | 7 | 0.9 | - | - | - | - | - | - | - | - | 0.5 | - | 1.6 | 2.0 | - | - | 4.5 | - | 0.2 | 0.3 | 0.6 | 0.04 |
| **C45** | Mesothelioma | 2 | 0.3 | - | - | - | - | - | - | - | 0.5 | - | - | - | - | - | - | 2.3 | - | 0.1 | 0.1 | 0.1 | 0.01 |
| **C46** | Kaposi sarcoma | - | - | - | - | - | - | - | - | - | - | - | - | - | - | - | - | - | - | - | - | - | - |
| **C47,C49** | Connective and soft tissue | 9 | 1.2 | - | 0.3 | - | - | - | 0.4 | 0.5 | 0.5 | - | - | 0.8 | 1.0 | 1.1 | - | - | 4.6 | 0.3 | 0.3 | 0.5 | 0.02 |
| **C50** | Breast | 2 | 0.3 | - | - | - | - | - | - | - | - | - | - | - | - | - | 1.5 | 2.3 | - | 0.1 | 0.1 | - | 0.02 |
| **C60** | Penis | 4 | 0.5 | - | - | - | - | - | - | - | - | - | - | - | 1.0 | 2.2 | - | - | 2.3 | 0.1 | 0.2 | 0.4 | 0.02 |
| **C61** | Prostate | 17 | 2.2 | - | - | - | - | - | - | - | - | - | - | - | 2.0 | 3.4 | 3.1 | 9.0 | 13.8 | 0.5 | 0.8 | 0.7 | 0.09 |
| **C62** | Testis | 2 | 0.3 | - | - | - | - | - | - | - | - | - | 0.7 | - | 1.0 | - | - | - | - | 0.1 | 0.1 | 0.3 | 0.01 |
| **C63** | Other male genital organs | 1 | 0.1 | - | - | - | - | - | - | - | - | - | - | - | 1.0 | - | - | - | - | - | - | 0.1 | - |
| **C64** | Kidney | 11 | 1.4 | - | - | - | 0.3 | 0.3 | - | - | - | 1.1 | 2.0 | 0.8 | 2.0 | - | 1.5 | - | - | 0.4 | 0.4 | 1.0 | 0.04 |
| **C65** | Renal pelvis | - | - | - | - | - | - | - | - | - | - | - | - | - | - | - | - | - | - | - | - | - | - |
| **C66** | Ureter | 1 | 0.1 | - | - | - | - | - | - | - | - | - | 0.7 | - | - | - | - | - | - | - | - | 0.1 | - |
| **C67** | Bladder | 14 | 1.8 | - | - | - | - | - | - | - | 0.5 | - | 0.7 | 1.6 | - | 1.1 | 1.5 | 9.0 | 9.2 | 0.5 | 0.6 | 0.6 | 0.07 |
| **C68** | Other urinary organs | - | - | - | - | - | - | - | - | - | - | - | - | - | - | - | - | - | - | - | - | - | - |
| **C69** | Eye | 1 | 0.1 | - | - | - | - | - | - | - | - | - | - | - | - | - | - | - | 2.3 | - | - | - | - |
| **C70-72** | Brain, nervous system | 17 | 2.2 | - | - | 0.3 | - | - | - | 0.5 | 1.4 | 1.1 | 0.7 | 0.8 | 5.9 | 1.1 | 1.5 | - | - | 0.5 | 0.6 | 1.7 | 0.07 |
| **C73** | Thyroid | 10 | 1.3 | - | - | - | - | - | - | - | - | - | 0.7 | - | 1.0 | 2.2 | 1.5 | 4.5 | 6.9 | 0.3 | 0.4 | 0.5 | 0.05 |
| **C74** | Adrenal gland | - | - | - | - | - | - | - | - | - | - | - | - | - | - | - | - | - | - | - | - | - | - |
| **C75** | Other endocrine | - | - | - | - | - | - | - | - | - | - | - | - | - | - | - | - | - | - | - | - | - | - |
| **C81** | Hodgkin disease | 1 | 0.1 | - | 0.3 | - | - | - | - | - | - | - | - | - | - | - | - | - | - | - | - | - | - |
| **C82-85,C96** | Non-Hodgkin lymphoma | 13 | 1.7 | - | - | 0.3 | - | 0.3 | 0.4 | - | - | - | 0.7 | 1.6 | - | 4.5 | - | 2.3 | 4.6 | 0.4 | 0.5 | 1.0 | 0.05 |
| **C88** | Immunoproliferative diseases | - | - | - | - | - | - | - | - | - | - | - | - | - | - | - | - | - | - | - | - | - | - |
| **C90** | Multiple myeloma | 7 | 0.9 | - | - | - | - | - | - | - | - | - | 0.7 | - | - | 2.2 | - | 2.3 | 6.9 | 0.2 | 0.3 | 0.4 | 0.03 |
| **C91** | Lymphoid leukaemia | 1 | 0.1 | - | - | - | - | - | - | - | - | - | - | - | - | - | 1.5 | - | - | - | - | - | 0.01 |
| **C92-94** | Myeloid leukaemia | 13 | 1.7 | 0.4 | 0.3 | - | 0.3 | - | - | 1.4 | 1.0 | 1.1 | 0.7 | - | - | - | 1.5 | - | 2.3 | 0.4 | 0.4 | 0.5 | 0.03 |
| **C95** | Leukaemia unspecified | 9 | 1.2 | - | - | 0.3 | 0.3 | - | 0.4 | 0.5 | 1.0 | - | - | - | - | 2.2 | - | 2.3 | - | 0.3 | 0.3 | 0.5 | 0.03 |
| Myeloproliferative disorders | | - | - | - | - | - | - | - | - | - | - | - | - | - | - | - | - | - | - | - | - | - | - |
| Myelodysplastic syndromes | | - | - | - | - | - | - | - | - | - | - | - | - | - | - | - | - | - | - | - | - | - | - |
| Other and unspecified* | | 73 | 9.6 | 1.6 | 0.3 | 0.3 | 0.8 | 0.6 | 0.4 | 0.9 | 2.9 | 3.2 | 2.7 | 4.8 | 6.9 | 7.8 | 15.4 | 18.1 | 11.5 | 2.3 | 2.9 | 4.4 | 0.33 |
| **Total** | | **759** | **100.0** | **2.9** | **1.2** | **1.1** | **2.8** | **3.9** | **3.4** | **10.5** | **15.7** | **20.9** | **34.0** | **55.6** | **88.6** | **99.5** | **152.1** | **228.3** | **273.7** | **24.4** | **31.2** | **46.9** | **3.60** |

**Other and Unspecified sites include ICD codes: C48, C76, C77, and C80*
