# Supplementary material for: Cancer Risk in Nepal: An Analysis from Population-Based Cancer Registry of Urban, Suburban, and Rural Regions
Source: J Cancer Epidemiol. 2024 Jul 10;2024:4687221. doi: 10.1155/2024/4687221 (PMC11949594; doi:10.1155/2024/4687221)
Supplement: Supplementary 4 — S4_Table: cancer mortality among women. [file 4687221.f4.docx]

**Cancer Risk in Nepal: An Analysis from Population-Based Cancer Registry of Urban, Sub-urban and Rural Regions**

Corresponding Author:

Uma Kafle Dahal (dahaluma1@gmail.com)

Gehanath Baral (baraldr@gmail.com)

Supplementary Table 4 (S4_Table)

This is the standard registry table based on the 2019 data created by the author/s

| **S4_Table: Estimation of Cancer Mortality Cases, Relative Proportions (RP), Age Specific Rate, Crude Mortality Rate (CR) Age Standardized (world) Rate (AAR), Truncated Rate (aged 35-65), and Cumulative Risk Percent (0-74) Among Women** | | | | | | | | | | | | | | | | | | | | | | | |
| --- | --- | --- | --- | --- | --- | --- | --- | --- | --- | --- | --- | --- | --- | --- | --- | --- | --- | --- | --- | --- | --- | --- | --- |
| **ICD (10th)** | **SITES** | **Total** | **(%)** | **Age Group (years)** | | | | | | | | | | | | | | | | **CR** | **AAR** | **TR** | **Cum Risk % (0-74)** |
|  |  |  |  | **0-4** | **5-9** | **10-14** | **15-19** | **20-24** | **25-29** | **30-34** | **35-39** | **40-44** | **45-49** | **50-54** | **55-59** | **60-64** | **65-69** | **70-74** | **75+** |  |  |  |  |
| **C00** | Lip | - | - | - | - | - | - | - | - | - | - | - | - | - | - | - | - | - | - | - | - | - | - |
| **C01-02** | Tongue | 7 | 1.0 | - | - | - | - | - | - | 0.4 | - | - | 0.7 | - | - | 1.1 | 3.2 | 2.3 | 2.0 | 0.2 | 0.3 | 0.3 | 0.04 |
| **C03-06** | Mouth | 7 | 1.0 | - | - | - | - | - | - | - | - | - | 0.7 | 2.6 | 1.0 | 1.1 | - | 2.3 | - | 0.2 | 0.3 | 0.8 | 0.04 |
| **C07-08** | Salivary glands | - | - | - | - | - | - | - | - | - | - | - | - | - | - | - | - | - | - | - | - | - | - |
| **C09** | Tonsil | - | - | - | - | - | - | - | - | - | - | - | - | - | - | - | - | - | - | - | - | - | - |
| **C10** | Other oropharynx | 1 | 0.1 | - | - | - | - | - | - | - | - | - | - | - | - | - | 1.6 | - | - | - | - | - | 0.01 |
| **C11** | Nasopharynx | 1 | 0.1 | - | - | - | - | - | - | - | - | - | - | - | - | - | 1.6 | - | - | - | - | - | 0.01 |
| **C12-13** | Hypopharynx | 2 | 0.3 | - | - | - | - | - | - | - | - | - | - | - | - | 1.1 | - | - | 2.0 | 0.1 | 0.1 | 0.1 | 0.01 |
| **C14** | Pharynx unspecified | - | - | - | - | - | - | - | - | - | - | - | - | - | - | - | - | - | - | - | - | - | - |
| **C15** | Oesophagus | 9 | 1.3 | - | - | - | - | - | - | 0.8 | - | - | - | 0.9 | 1.0 | - | - | 4.6 | 6.0 | 0.3 | 0.3 | 0.3 | 0.04 |
| **C16** | Stomach | 29 | 4.3 | - | - | - | - | - | 0.3 | - | 1.3 | - | 1.4 | 2.6 | 5.1 | 4.5 | 4.8 | 4.6 | 12.0 | 0.9 | 1.2 | 2.2 | 0.12 |
| **C17** | Small intestine | 1 | 0.1 | - | - | - | - | 0.3 | - | - | - | - | - | - | - | - | - | - | - | - | - | - | - |
| **C18** | Colon | 23 | 3.4 | - | - | - | - | - | 0.3 | 0.4 | 1.3 | 0.5 | 1.4 | 2.6 | 1.0 | - | 3.2 | 9.2 | 10.0 | 0.7 | 0.9 | 1.2 | 0.10 |
| **C19-20** | Rectum | 15 | 2.2 | - | - | - | - | 0.6 | 1.0 | 0.4 | 0.4 | 0.5 | - | 0.9 | 1.0 | 1.1 | 1.6 | 2.3 | 4.0 | 0.5 | 0.5 | 0.6 | 0.05 |
| **C21** | Anus | 2 | 0.3 | - | - | - | - | - | - | - | - | - | - | - | 1.0 | - | 1.6 | - | - | 0.1 | 0.1 | 0.1 | 0.01 |
| **C22** | Liver | 21 | 3.1 | - | - | - | - | - | - | - | - | 1.6 | 0.7 | 1.7 | 7.2 | 2.2 | 6.4 | - | 4.0 | 0.7 | 0.9 | 1.9 | 0.10 |
| **C23-24** | Gallbladder etc. | 84 | 12.6 | - | - | - | - | - | 0.3 | 0.4 | 2.1 | 1.6 | 4.8 | 6.9 | 9.3 | 13.4 | 30.2 | 20.8 | 20.1 | 2.7 | 3.5 | 5.7 | 0.45 |
| **C25** | Pancreas | 14 | 2.1 | - | - | - | - | - | - | - | - | - | 1.4 | 0.9 | 5.1 | - | 3.2 | - | 8.0 | 0.4 | 0.6 | 1.1 | 0.05 |
| **C30-31** | Nose, sinuses etc. | - | - | - | - | - | - | - | - | - | - | - | - | - | - | - | - | - | - | - | - | - | - |
| **C32** | Larynx | 3 | 0.4 | - | - | - | - | - | - | - | 0.4 | - | - | 0.9 | - | - | 1.6 | - | - | 0.1 | 0.1 | 0.2 | 0.01 |
| **C33-34** | Trachea, bronchus and lung | 114 | 17.1 | - | - | - | - | 0.3 | 0.3 | 0.8 | - | 1.6 | 6.1 | 7.8 | 9.3 | 24.5 | 27.0 | 37.0 | 50.2 | 3.6 | 4.9 | 7.1 | 0.57 |
| **C37-38** | Other thoracic organs | - | - | - | - | - | - | - | - | - | - | - | - | - | - | - | - | - | - | - | - | - | - |
| **C40-41** | Bone | 9 | 1.3 | 0.4 | - | 0.3 | 0.3 | 0.3 | - | - | - | 0.5 | - | - | 1.0 | 1.1 | 1.6 | 2.3 | - | 0.3 | 0.3 | 0.4 | 0.04 |
| **C43** | Melanoma of skin | 1 | 0.1 | - | - | - | - | - | - | - | - | - | - | - | - | 1.1 | - | - | - | - | - | 0.1 | 0.01 |
| **C44** | Other skin | 4 | 0.6 | - | - | - | - | - | - | - | - | 0.5 | - | - | - | - | 1.6 | - | 4.0 | 0.1 | 0.2 | 0.1 | 0.01 |
| **C45** | Mesothelioma | 1 | 0.1 | - | - | - | - | - | - | - | - | - | - | - | - | - | 1.6 | - | - | - | - | - | 0.01 |
| **C46** | Kaposi sarcoma | - | - | - | - | - | - | - | - | - | - | - | - | - | - | - | - | - | - | - | - | - | - |
| **C47,C49** | Connective and soft tissue | 7 | 1.0 | 0.4 | - | - | - | 0.6 | - | - | - | 0.5 | - | - | - | 1.1 | - | 2.3 | 2.0 | 0.2 | 0.3 | 0.2 | 0.03 |
| **C50** | Breast | 82 | 12.3 | - | - | - | - | 0.3 | 0.6 | 0.8 | 3.0 | 8.6 | 6.1 | 10.4 | 16.5 | 5.6 | 8.0 | 6.9 | 8.0 | 2.6 | 3.1 | 7.9 | 0.33 |
| **C51** | Vulva | 5 | 0.7 | - | - | - | - | - | - | - | - | - | - | 0.9 | 1.0 | 1.1 | - | 2.3 | 2.0 | 0.2 | 0.2 | 0.4 | 0.03 |
| **C52** | Vagina | 2 | 0.3 | - | - | - | - | - | - | - | - | - | 0.7 | - | 1.0 | - | - | - | - | 0.1 | 0.1 | 0.3 | 0.01 |
| **C53** | Cervix uteri | 61 | 9.1 | - | - | - | - | 0.3 | - | - | 1.7 | 3.8 | 5.4 | 6.0 | 6.2 | 10.0 | 11.1 | 13.9 | 12.0 | 1.9 | 2.5 | 5.2 | 0.29 |
| **C54** | Corpus uteri | 4 | 0.6 | - | - | - | - | - | - | - | 0.4 | - | - | - | 1.0 | 2.2 | - | - | - | 0.1 | 0.2 | 0.5 | 0.02 |
| **C55** | Uterus unspecified | 9 | 1.3 | - | - | - | - | - | - | 0.4 | - | 1.1 | 0.7 | 0.9 | - | - | 1.6 | 2.3 | 4.0 | 0.3 | 0.3 | 0.5 | 0.03 |
| **C56** | Ovary | 30 | 4.5 | - | - | - | - | - | 0.3 | 0.8 | 0.8 | 1.1 | 2.7 | 1.7 | 3.1 | 2.2 | 6.4 | 4.6 | 12.0 | 1.0 | 1.2 | 1.9 | 0.12 |
| **C57** | Other female genital organs | - | - | - | - | - | - | - | - | - | - | - | - | - | - | - | - | - | - | - | - | - | - |
| **C58** | Placenta | - | - | - | - | - | - | - | - | - | - | - | - | - | - | - | - | - | - | - | - | - | - |
| **C64** | Kidney | 6 | 0.9 | - | - | - | - | - | - | - | - | - | - | 1.7 | 1.0 | - | 1.6 | 2.3 | 2.0 | 0.2 | 0.3 | 0.4 | 0.03 |
| **C65** | Renal pelvis | 2 | 0.3 | - | - | - | - | - | - | - | - | - | - | 0.9 | - | - | - | - | 2.0 | 0.1 | 0.1 | 0.1 | - |
| **C66** | Ureter | - | - | - | - | - | - | - | - | - | - | - | - | - | - | - | - | - | - | - | - | - | - |
| **C67** | Bladder | 6 | 0.9 | - | - | - | - | - | - | - | - | - | 0.7 | 0.9 | - | 3.3 | - | - | 2.0 | 0.2 | 0.3 | 0.7 | 0.02 |
| **C68** | Other urinary organs | - | - | - | - | - | - | - | - | - | - | - | - | - | - | - | - | - | - | - | - | - | - |
| **C69** | Eye | 1 | 0.1 | 0.4 | - | - | - | - | - | - | - | - | - | - | - | - | - | - | - | - | 0.1 | - | - |
| **C70-72** | Brain, nervous system | 13 | 1.9 | - | 0.3 | - | 0.3 | - | - | 0.8 | 0.8 | 0.5 | 0.7 | 0.9 | 1.0 | 1.1 | 1.6 | - | 2.0 | 0.4 | 0.4 | 0.8 | 0.04 |
| **C73** | Thyroid | 5 | 0.7 | - | - | - | - | - | - | - | - | 0.5 | - | - | 1.0 | 3.3 | - | - | - | 0.2 | 0.2 | 0.7 | 0.02 |
| **C74** | Adrenal gland | - | - | - | - | - | - | - | - | - | - | - | - | - | - | - | - | - | - | - | - | - | - |
| **C75** | Other endocrine | - | - | - | - | - | - | - | - | - | - | - | - | - | - | - | - | - | - | - | - | - | - |
| **C81** | Hodgkin disease | - | - | - | - | - | - | - | - | - | - | - | - | - | - | - | - | - | - | - | - | - | - |
| **C82-85,C96** | Non-Hodgkin lymphoma | 12 | 1.8 | - | - | - | - | - | - | 1.1 | - | 1.1 | - | 0.9 | - | 2.2 | 4.8 | - | 2.0 | 0.4 | 0.4 | 0.6 | 0.05 |
| **C88** | Immunoproliferative diseases | - | - | - | - | - | - | - | - | - | - | - | - | - | - | - | - | - | - | - | - | - | - |
| **C90** | Multiple myeloma | 1 | 0.1 | - | - | - | - | - | - | - | - | - | - | - | - | - | - | 2.3 | - | - | - | - | 0.01 |
| **C91** | Lymphoid leukaemia | 1 | 0.1 | - | - | - | 0.3 | - | - | - | - | - | - | - | - | - | - | - | - | - | - | - | - |
| **C92-94** | Myeloid leukaemia | 6 | 0.9 | - | - | - | 0.3 | - | - | 1.1 | - | - | - | - | - | - | 1.6 | 2.3 | - | 0.2 | 0.2 | - | 0.03 |
| **C95** | Leukaemia unspecified | 3 | 0.4 | - | - | 0.3 | - | 0.3 | - | - | - | - | - | - | - | - | - | - | 2.0 | 0.1 | 0.1 | - | - |
| Myeloproliferative disorders | | - | - | - | - | - | - | - | - | - | - | - | - | - | - | - | - | - | - | - | - | - | - |
| Myelodysplastic syndromes | | 1 | 0.1 | - | - | - | - | - | - | - | - | - | - | - | - | - | - | - | 2.0 | - | - | - | - |
| Other and unspecified* | | 63 | 9.4 | 0.4 | - | - | - | 0.3 | - | 0.4 | 1.3 | 3.2 | 6.1 | 6.9 | 5.1 | 7.8 | 19.1 | 11.6 | 10.0 | 2.0 | 2.6 | 4.8 | 0.31 |
| **Total** | | **668** | **100** | **1.8** | **0.3** | **0.5** | **1.3** | **3.3** | **3.2** | **8.3** | **13.6** | **27.6** | **40.2** | **59.5** | **79.3** | **91.2** | **146.4** | **136.3** | **186.7** | **21.3** | **27.0** | **47.3** | **3.02** |

**Other and Unspecified sites include ICD codes: C26, C76, C77 and C80*
