# Supplementary material for: Cancer Risk in Nepal: An Analysis from Population-Based Cancer Registry of Urban, Suburban, and Rural Regions
Source: J Cancer Epidemiol. 2024 Jul 10;2024:4687221. doi: 10.1155/2024/4687221 (PMC11949594; doi:10.1155/2024/4687221)
Supplement: Supplementary 5 — S5_Table: five most common cancers in urban, suburban, and rural regions of the country. [file 4687221.f5.docx]

**Cancer Risk in Nepal: An Analysis from Population-Based Cancer Registry of Urban, Sub-urban and Rural Regions**

Corresponding Author

Uma Kafle Dahal (dahaluma1@gmail.com)

Gehanath Baral ([baraldr@gmail.com](mailto:baraldr@gmail.com))

Supplementary Table 5 (**S5-Table)**

| **S5-Table:** Five most common cancers in urban, suburban, and rural regions | | | | | | | | | | | | |
| --- | --- | --- | --- | --- | --- | --- | --- | --- | --- | --- | --- | --- |
| **Top 5 most frequent cancers in urban, suburban, and rural regions in 2019, Men** | | | | | | | | | | | | |
|  | **Urban** | | | | **Suburban** | | | | **Rural** | | | |
| **Rank** | **Cancer site** | **Number of cases** | **%** | **AAR** | **Cancer site** | **Number of cases** | **%** | **AAR** | **Cancer site** | **Number of cases** | **%** | **AAR** |
| 1st | Lung | 165 | 17.6 | 16.7 | Mouth | 75 | 13 | 6.2 | Lung | 8 | 18.6 | 9.7 |
| 2nd | Stomach | 86 | 9.3 | 8.3 | Lung | 54 | 9.3 | 4.4 | Stomach | 5 | 11.6 | 6.1 |
| 3rd | Prostate | 49 | 5.3 | 4.9 | Stomach | 37 | 6.4 | 3.3 | Urinary Bladder | 3 | 7.0 | 4.4 |
| 4th | Colon | 51 | 5.5 | 4.5 | Gallbladder | 34 | 6 | 2.9 | Thyroid | 3 | 7.0 | 3.5 |
| 5th | Urinary bladder | 43 | 4.6 | 4.2 | Liver | 35 | 5.9 | 2.9 | Prostate | 3 | 7.0 | 3.2 |
| **Top 5 most frequent cancers in urban, suburban, and rural regions in 2019, Women** | | | | | | | | | | | | |
|  | **Urban** | | | | **Suburban** | | | | **Rural** | | | |
| **Rank** | **Cancer site** | **Number of cases** | **%** | **AAR** | **Cancer site** | **Number of cases** | **%** | **AAR** | **Cancer site** | **Number of cases** | **%** | **AAR** |
| 1st | Breast | 221 | 22.4 | 17.5 | Breast | 103 | 16.9 | 8 | Lung | 10 | 22.2 | 10.9 |
| 2nd | Lung | 122 | 11.3 | 11 | Cervix | 89 | 14.6 | 7.4 | Breast | 7 | 15.6 | 7.7 |
| 3rd | Cervix | 98 | 9.1 | 8.3 | Gallbladder | 71 | 11.7 | 5.7 | Cervix | 6 | 13.3 | 7 |
| 4th | Gallbladder | 72 | 6.7 | 6.7 | Lung | 49 | 8 | 4.4 | Ovary | 2 | 4.4 | 2.2 |
| 5th | Ovary | 71 | 6.6 | 5.5 | Ovary | 27 | 4.4 | 2.2 | Stomach | 2 | 4.4 | 2.2 |
| % is the relative proportion of cases registered in respective regions, and AAR is the adjusted Rate per 100,000 population | | | | | | | | | | | | |
