# Supplementary material for: Cancer Risk in Nepal: An Analysis from Population-Based Cancer Registry of Urban, Suburban, and Rural Regions
Source: J Cancer Epidemiol. 2024 Jul 10;2024:4687221. doi: 10.1155/2024/4687221 (PMC11949594; doi:10.1155/2024/4687221)
Supplement: Supplementary 6 — S6_Table: quality indicators of cases registered in urban, suburban, and rural regions. [file 4687221.f6.docx]

**Cancer Risk in Nepal: An Analysis from Population-Based Cancer Registry of Urban, Sub-urban and Rural Regions**

Corresponding Author:

Uma Kafle Dahal (dahaluma1@gmail.com)

Gehanath Baral ([baraldr@gmail.com](mailto:baraldr@gmail.com))

Supplementary Table 6 (S6_Table)

**S6_Table: Quality indicators of cases registered in urban, suburban, and rural regions (in percent)**

| **Registry Sites** | **Number of total cases registered** | **Clinical Investigation (CT, MRI, USG, etc.)** | **Clinical Note** | **Microscopic**  **Verification (Cytology and Histopathology** | **DCO** | **Others** | | | **Total (%)** | **Mortality -to-Incidence ratio** |
| --- | --- | --- | --- | --- | --- | --- | --- | --- | --- | --- |
|  |  |  |  |  |  | **Verbal Information** | **Tumor Marker** | **Hospital Record** |  |  |
| Urban | 2019 | 2.4 | 4.9 | 91.7 | 0.1 | 0.6 | 0.1 | 0 | 100 | 42 |
| Suburban | 1188 | 4.5 | 7.1 | 51.6 | 2.8 | 31.5 | 0 | 2.5 | 100 | 44.8 |
| Rural | 88 | 3.4 | 4.5 | 60.2 | 2.3 | 22.7 | 0 | 6.8 | 100 | 53.4 |
| **Total** | **3295** | **3.3** | **5.7** | **76.4** | **1.1** | **12.4** | **0.1** | **1.1** | **100** | **43.3** |
